# C16-siRNAs in Focus: Development of ALN-APP, a Promising RNAi-Based Therapeutic for Alzheimer’s Disease

**DOI:** 10.3390/ph19010026

**Published:** 2025-12-22

**Authors:** Ricardo Titze-de-Almeida, Guilherme de Melo Oliveira Gomes, Tayná Cristina dos Santos, Simoneide Souza Titze-de-Almeida

**Affiliations:** 1 Technology for Gene Therapy Laboratory, ASS 128, ICC Sul, University of Brasília, Campus Darcy Ribeiro, FAV, Brasília 70910-900, DF, Brazil; 2 Research Center for Major Themes—Neurodegenerative Disorders Division, University of Brasília, Brasília 70910-900, DF, Brazil

**Keywords:** gene therapy, RNA interference, β-amyloid, Alzheimer’s disease, C16-siRNA

## Abstract

This review examines a small interfering RNA (siRNA) designed for intrathecal (IT) injection, which reduces the formation of amyloid beta precursor protein (APP), a critical factor in the pathology of Alzheimer’s disease (AD). The siRNA, designated ALN-APP, incorporates a 16-carbon chain (C16-siRNA) to enhance its delivery to the central nervous system (CNS) while leveraging advancements in specificity and duration of action based on previously approved drugs by the Food and Drug Administration. The development of ALN-APP involved a comprehensive analysis of the optimal carbon chain length and its conjugation position to the siRNA. Preclinical studies conducted on male Sprague Dawley rats, mice, and non-human primates (NHPs) demonstrated the efficacy of ALN-APP. In rats, an IT injection of C16-siRNAs at a concentration of 30 mg/mL, delivering a dose of 0.9 mg, resulted in cranial distribution via cerebrospinal fluid and led to a 75% reduction in copper-zinc superoxide dismutase 1 (SOD1) mRNA levels. These effects were dose-dependent and persisted for three months across multiple brain regions. Furthermore, studies in NHPs indicated that soluble APP levels were reduced to below 25%, sustained for two months. In the cerebrovascular amyloid Nos2^−/−^ (CVN) mouse model of AD, administration of 120 µg of siRNA via the intracerebroventricular route produced reductions in APP expression, with mRNA levels remaining suppressed for 60 days in the ventral cortex. Indeed, ALN-APP controlled neuropathology in 5xFAD mice by significantly reducing amyloid levels and brain neuroinflammation, with improved behaviors in the elevated plus maze. Following these promising results in animal models, ALN-APP advanced to a Phase 1 trial, designated ALN-APP-001, which assessed its safety and efficacy in 12 participants with early-onset Alzheimer’s disease (EOAD). Initial findings revealed a 55% reduction in soluble APPα and a 69% reduction in APPβ by day 15. These exploratory findings require further validation with larger cohorts and proper statistical analysis. In a subsequent cohort of 36 patients, administration of the 75 mg dose via IT injection led to mean reductions of 61.3% in soluble APPα (sAPPα) and 73.5% in soluble APPβ (sAPPβ) after one month. These silencing effects persisted for six months and were associated with important decreases in Aβ42 and Aβ40 levels. These results highlight the potential of ALN-APPs to address Alzheimer’s pathology while maintaining a favorable safety profile. Whether ALN-APP succeeds in further clinical trials, key challenges include ensuring accessibility and affordability due to treatment costs, the need for specialized intrathecal administration, and establishing infrastructure for large-scale production of siRNAs. In conclusion, advancements in ALN-APP represent a promising strategy to reduce beta-amyloid formation in AD, with substantial biomarker reductions suggesting potential disease-modifying effects. Continued development may pave the way for innovative treatments for neurodegenerative diseases.

## 1. Introduction

Dementia has increasingly become a focal point in scientific research and public health initiatives. This growing attention is driven by the condition’s rising prevalence among the aging global population and its profound impact on affected individuals and their families [1]. Currently, around 50 million individuals worldwide are diagnosed with dementia, a number that is projected to triple by 2050 [2]. Alzheimer’s disease (AD) is the most common form of dementia globally. Despite significant progress in understanding the brain pathology associated with AD, treating this complex disorder remains challenging [1,3].

The incidence of AD is influenced by non-modifiable risk factors such as advancing age, female sex, lower educational attainment, and genetic predisposition, particularly the presence of the APOE (Apolipoprotein E) ε4 allele. Modifiable factors include cardiovascular conditions (such as hypertension, diabetes, hypercholesterolemia), lifestyle elements (including adherence to a Mediterranean diet, physical activity, cognitive engagement), and environmental exposures (for example, air pollution). Other contributing factors to AD risk include depression, obesity, subjective cognitive decline, neurodegenerative markers visible on imaging, and co-pathologies [4,5,6,7,8,9,10,11,12].

The pathological features of AD are primarily characterized by the accumulation of β-amyloid (Aβ) plaques and neurofibrillary tangles (NFTs) composed of tau protein in the brain. Additional significant changes include an increase in reactive oxygen species, the proliferation of glial cells, impaired insulin sensitivity, and modifications in the microbiome [13]. These characteristics have been extensively studied due to their critical importance for diagnostic and potential therapeutic applications [14]. Amyloid plaques are extracellular deposits of Aβ peptides that follow a predictable regional progression, starting in the neocortex and advancing to other brain areas [15,16,17,18,19].

Under normal physiological conditions, Aβ exists in a balance between production and clearance. Several mechanisms aid in clearing Aβ from the brain, including non-enzymatic pathways, such as its transport across blood vessel walls into the bloodstream, and enzymatic pathways involving neprilysin and insulin-degrading enzymes [20,21]. Cerebral Aβ is transported across the blood–brain barrier through scavenger receptors such as lipoprotein receptor-related protein 1 (LRP1) and very low-density lipoprotein receptor (VLDLR), which facilitate its clearance into the bloodstream in the human brain. Sequester proteins have been shown to enhance the binding affinity of Aβ to these scavenger receptors [22], stabilizing monomeric Aβ and inhibiting its aggregation [23]. Aβ is primarily present in two forms: Aβ40 and Aβ42. The main difference between them lies in the two extra residues at the C-terminus in the Aβ42, which alter its metabolism, physiological functions, toxicities, and aggregation mechanism [24,25]. Among these forms, Aβ42 is more toxic and more strongly associated with the development of AD-related pathologies than Aβ40 [22]. Growing evidence establishes that Aβ42 begins to accumulate in the brain many years, or even decades, before the clinical symptoms of AD appear [26].

While amyloid precursor protein (APP) has long been considered central to AD pathology, recent evidence indicates that neuronal autolysosome acidification declines months before Aβ deposition [27], suggesting that Aβ accumulation may represent a consequence rather than a cause of AD. This perspective helps explain the limited efficacy of Aβ-targeted therapies. Neuronal autophagy, essential for proteostasis, depends on retrograde transport of autophagosomes to the soma for fusion with lysosomes, forming acidified autolysosomes [28]. In AD, this process is disrupted, leading to autophagic vacuole (AV) accumulation [29,30]. Impairment arises through defective initiation, impaired AV transport, and lysosomal degradation failure [31,32]. Reduced Beclin-1 expression compromises autophagosome formation and correlates with Aβ buildup [33,34]. Aβ oligomers and hyperphosphorylated tau further hinder axonal transport by destabilizing microtubules [35,36]. Presenilin-1 (PS1) mutations exacerbate pathology by impairing v-ATPase–dependent lysosomal acidification while elevating lysosomal pH and promoting Aβ42 accumulation [30,37,38]. Additionally, ApoE4—the major genetic risk factor for sporadic AD—disrupts lysosomal homeostasis and enhances Aβ42 production, contributing to neuronal death [39]. This emerging view of autophagy dysfunction as an early and potentially causal event in AD pathogenesis broadens the understanding of disease mechanisms and highlights novel therapeutic targets beyond Aβ clearance.

Currently, the treatment landscape for AD is limited, with only two major classes of drugs available: cholinesterase inhibitors and N-methyl D-aspartate (NMDA) antagonists. These medications primarily provide symptomatic relief and do not cure or prevent the disease. Furthermore, they often come with undesirable side effects, highlighting the significant limitations of current therapies [40,41,42]. Monoclonal antibodies (mAbs) have recently gained attention as potential disease-modifying therapies for AD. The Food and Drug Administration (FDA) has approved mAbs like aducanumab and lecanemab, which promise to potentially modify disease progression. However, the long-term efficacy and safety of these treatments remain uncertainties that require further investigation [43,44,45].

Given the existing limitations of current therapies, including their focus on symptom management and associated side effects, there is a pressing need to explore new treatment strategies and targets. This ongoing search for safer and more effective interventions is critical to improving the outcomes for individuals affected by AD.

The current review presents a narrative on the evolution of siRNA biotechnology, beginning with an overview of the first RNAi drugs, patisiran and givosiran, which enabled effective hepatic delivery. This progress has paved the way for the C16 siRNA mivelsiran (ALN-APP), our primary focus. We offer an updated analysis of the latest findings from preclinical studies involving ALN-APP in rodent models and non-human primates, including the use of the IT route of administration, the effects on brain neuropathology and behavior, and some preliminary Phase I clinical data.

## 2. siRNAs—Concept and Mechanism of Action

Small interfering RNAs (siRNAs) are short, double-stranded RNA molecules that play a crucial role in regulating gene expression through a process known as RNA interference (RNAi). These molecules comprise 19 to 23 ribonucleotides and exhibit specificity in binding to messenger RNA (mRNA) via Watson–Crick base pairing [46].

**Figure 1:**
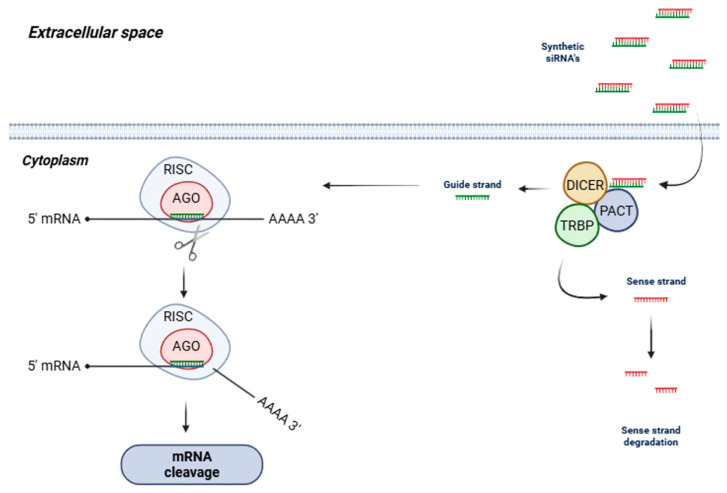
The siRNA pathway mediating post-transcriptional gene silencing. RNA interference (RNAi) is a highly conserved cellular pathway allowing targeted gene silencing at the post-transcriptional level. Synthetic siRNA duplexes consist of a guide (green) and a sense (red) strand. Once inside the cytoplasm, the duplex is recognized by a protein complex that includes the ribonuclease Dicer, the transactivation response RNA-binding protein (TRBP), and the protein activator of the interferon-induced protein kinase (PACT). This complex facilitates the removal and degradation of the sense strand, while the guide strand is retained and incorporated into the RNA-induced silencing complex (RISC). The mature RISC, which includes Argonaute 2 (AGO2) and the guide strand, is directed by the guide strand to bind complementary sequences on target messenger RNAs (mRNAs). Upon binding, AGO2 catalyzes the cleavage of the target mRNA, resulting in sequence-specific degradation and subsequent gene silencing. Abbreviations: AGO2, Argonaute 2 enzyme; mRNA, messenger RNA; PACT, protein activator of the interferon-induced protein kinase; RISC, RNA-induced silencing complex; RNAi, RNA interference; siRNA, small interfering RNA; TRBP, HIV-1 transactivation response (TAR) RNA-binding protein.

The discovery of siRNAs and the elucidation of the RNAi process can be traced back to the groundbreaking research conducted by Andrew Fire and Craig Mello, who were awarded the Nobel Prize for their work in this area. They characterized the gene-silencing effects of short RNAs and introduced the term “RNA interference” in their seminal publication [47]. The mechanisms governing RNAi-mediated gene silencing are illustrated in Figure 1 and have been reviewed extensively in the literature [48,49,50,51,52,53].

Inside the cell, siRNA duplexes are first processed by the enzymes Dicer, TRBP, and PACT, which remove the sense strand. Then, the remaining guide strand is incorporated into a protein complex known as the RNA-induced silencing complex (RISC). This guide strand subsequently searches for complementary nucleotides in target mRNAs through a trial-and-error mechanism, forming hydrogen bonds with matching sequences [54,55]. Once bound, the RISC, assisted by the guide strand, executes cleavage of the targeted mRNA [56,57].

**Figure 2:**
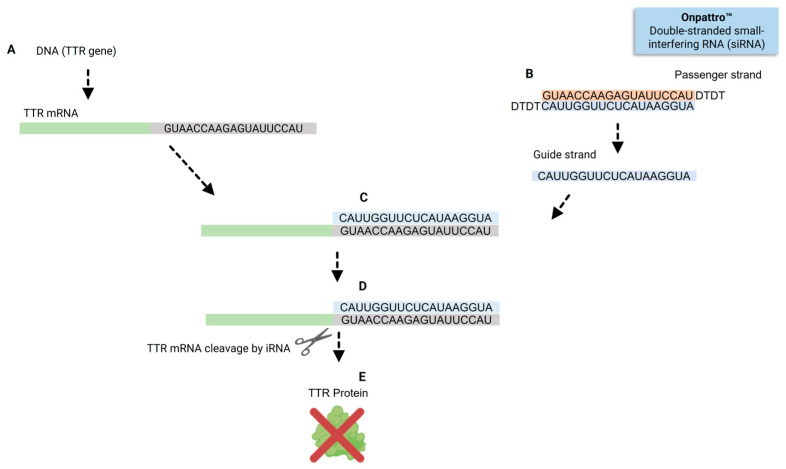
Mechanism of action and silencing effects of patisiran on TTR mRNA. (**A**) The TTR gene expresses the TTR mRNA that, in the 3′-UTR region, contains a sequence conserved across wild-type (wt) and all TTR variant forms. (**B**) Patisiran is a double-stranded siRNA composed of a passenger and guide strand with overhangs at the 3′ ends. (**C**) The passenger strand (in orange) is eliminated, and the guide strand (in blue, without overhangs) finds the complementary nucleotides in the 3′ UTR region of TTR mRNA (in gray) for base pairing. (**D**) The binding of patisiran to TTR mRNA induces the RISC to execute RNAi-mediated gene silencing by cleavage of this 3′ UTR region, and (**E**) synthesis of the TTR protein is suppressed. Abbreviations: siRNA, small-interfering RNA; mRNA, messenger RNA; iRNA, RNA interference; TTR, transthyretin; UTR, untranslated region; wt, wild-type.

siRNAs have successfully transitioned into clinical settings and are recognized as FDA-approved therapeutics. Patisiran, for instance, was the first siRNA therapeutic to complete all clinical development phases and receive regulatory approval for market entry. Patisiran’s nucleotide composition illustrates how siRNAs identify specific sequences in the mRNA to knock down, as shown in Figure 2. This drug operates as a short RNA molecule that selectively binds to and induces the cleavage of target mRNA through the RNAi pathway [58,59,60].

Patisiran is indicated for the treatment of hereditary transthyretin-mediated (hATTR) amyloidosis [60,61]. It represents a leading example among ten RNAi-based therapeutics that entered Phase II–III clinical trials beginning in 2017, and ultimately received approval from the FDA in the following year [48,61]. This innovative class of therapeutics represents a substantial advancement in pharmacology, introducing a unique mechanism of action based on sequence-specific gene silencing at the post-transcriptional level [48].

Following a similar mechanism of action used by patisiran, the biopharmaceutical company Alnylam has developed a second siRNA to treat porphyria, known as givosiran. This double-stranded siRNA knocks down ALAS1 mRNA [62,63].

## 3. A Brief Overview of Patisiran and Givosiran Biotechnology: The First Two FDA-Approved siRNAs Demonstrating Organ-Specific Delivery Viability

Biotechnology is inherently a multi-step, progressive process. The challenges surmounted by a specific product can indicate strategies that may be adapted for other products; this has been particularly evident in modifying the chemical structure of siRNAs’ nucleotides to enable sustained and more specific therapeutic effects. Another notable example is the encapsulation of siRNAs within dedicated lipid nanoparticles, which facilitated their delivery to the liver—a breakthrough achieved by patisiran. Additionally, givosiran employs a different delivery mechanism, utilizing a GalNAc molecule conjugated to the siRNA, enabling targeted delivery to the liver without the need for lipid nanoparticles [63].

These advancements illustrate the sequential steps in the evolution of siRNA biotechnology, underscoring its dynamic and progressive nature. The FDA approval of these drugs warrants a concise historical narrative to elucidate the biotechnological pathways of conjugating molecules to siRNAs, as these innovations have laid the groundwork for developing C16 siRNAs, which will be the primary focus of this review.

**Figure 3:**
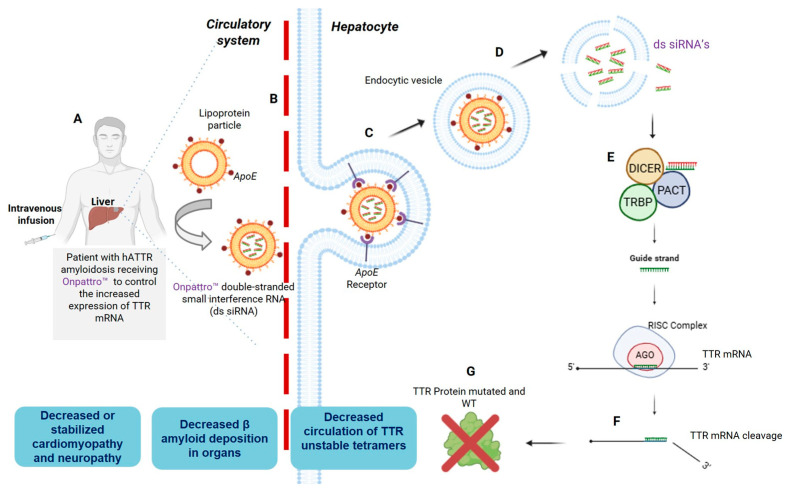
The mechanism of patisiran therapy for treating hereditary transthyretin amyloidosis (hATTR amyloidosis). Patisiran, the first FDA-approved siRNA therapeutic for hATTR amyloidosis, reduces transthyretin (TTR) expression through RNA interference (RNAi). (**A**) Patients receive patisiran via intravenous (IV) infusion. (**B**) Patisiran is encapsulated in lipid nanoparticles (LNPs), which are naturally opsonized by apolipoprotein E (ApoE) in the circulatory system. (**C**) The ApoE-coated LNPs bind to ApoE receptors on hepatocytes and are internalized via receptor-mediated endocytosis. (**D**) Within the acidic environment of endocytic vesicles, the ionizable lipids in the LNPs become protonated, acquiring a positive charge. This change promotes electrostatic interaction with the negatively charged endosomal membrane, leading to membrane fusion and cytoplasmic release of the double-stranded siRNA (ds-siRNA). (**E**) In the cytoplasm, the ds-siRNA is processed by a protein complex consisting of Dicer (a ribonuclease), TRBP (HIV-1 trans-activation response RNA-binding protein), and PACT (protein activator of the interferon-induced protein kinase). This complex removes the passenger (sense) strand, while the guide (antisense) strand is loaded into the RNA-induced silencing complex (RISC). (**F**) The Argonaute 2 (AGO2) enzyme within RISC directs the guide strand to bind complementary sequences in TTR mRNA, resulting in targeted cleavage and degradation of the transcript. (**G**) Suppression of TTR mRNA translation reduces the production of unstable TTR tetramers, thereby preventing their misfolding and aggregation into amyloid fibrils. This reduction in amyloid burden stabilizes or improves clinical manifestations such as neuropathy and cardiomyopathy. Abbreviations: AGO2, Argonaute 2 enzyme; ApoE, apolipoprotein E; ds-siRNA, double-stranded small interfering RNA; hATTR amyloidosis, hereditary transthyretin amyloidosis; IV, intravenous; LNPs, lipid nanoparticles; mRNA, messenger RNA; PACT, protein activator of the interferon-induced protein kinase; RISC, RNA-induced silencing complex; RNAi, RNA interference; siRNA, small interfering RNA; TRBP, HIV-1 trans-activation response (TAR) RNA-binding protein; TTR, transthyretin.

The first FDA-approved siRNA was patisiran. Its therapeutic efficacy heavily relies on its lipid nanoparticle (LNP) delivery system, which facilitates targeted delivery to hepatocytes (Figure 3). The LNPs used for patisiran are coated with ApoE, which mediates nanoparticles’ opsonization. This process allows the particles to traverse the fenestrated hepatic endothelium and bind specifically to ApoE receptors on the surface of hepatocytes. After receptor-mediated endocytosis, the LNPs are internalized, and the ionizable lipids within the particles are protonated in the acidic endosomal environment. This protonation induces fusion of the LNP membrane with the endosomal membrane, destabilizing the lipid structure and releasing free siRNAs into the cytoplasm [64,65].

Once in the cytoplasm, the siRNAs trigger a similar RNAi mechanism as described above. The guide strands interact with RISC and direct the complex to complementary TTR mRNA sequences. This interaction triggers RNAi-mediated degradation of the TTR transcript (Figure 3F). The specificity of this process is determined by the base-pairing between the siRNA guide strand and its target mRNA. For instance, the guide strand of patisiran, with the sequence 3′-CAUUGGUUCUCAUAAGGUA-5′, exhibits perfect complementarity to the TTR mRNA sequence 5′-GUAACCAAGAGUAUUCCAU-3′, as shown in Figure 2C. Notably, the 3′ terminal triplet “CAU” of the siRNA pairs precisely with the 5′ triplet “GUA” of the TTR mRNA, underscoring the critical importance of sequence complementarity for efficient RNAi-based gene silencing [66,67,68].

**Figure 4:**
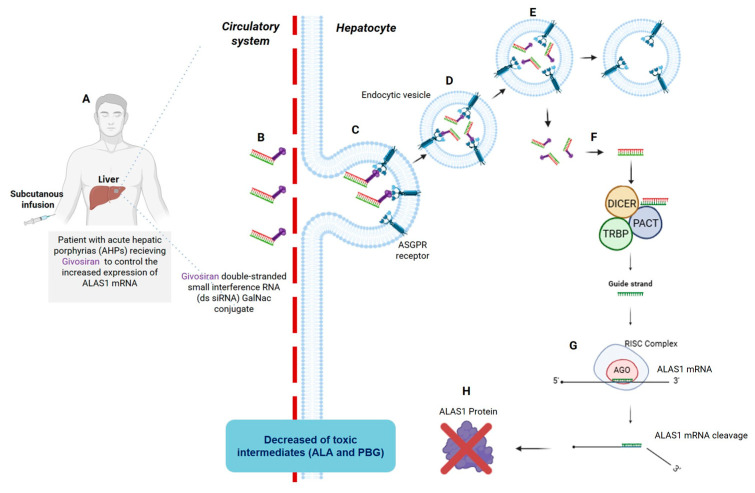
Givosiran therapy for reducing acute hepatic porphyria attacks (AHP). (**A**) Patients with AHP receive givosiran by subcutaneous injection. (**B**) Anti-ALAS1 siRNA contains an N-acetyl galactosamine (GalNac) molecule conjugated at the 3’-end of the passenger strand. (**C**) GalNac moiety binds to asialoglycoprotein receptor (ASGPR) on hepatocytes. (**D**) An endocytic vesicle is formed and internalizes the siRNA molecule into the cell cytoplasm. (**E**) siRNA GalNac conjugated is delivered in the cytoplasm. (**F**) The siRNA molecule forms a complex with Dicer, TRBP, and PACT proteins, which will eliminate the passenger strand. (**G**) The remaining siRNA guide strand loaded in the RISC binds to complementary sequences in ALAS1 mRNA, allowing the AGO enzyme to execute the cleavage of ALAS1 mRNA. (**H**) Knockdown of ALAS1 expression prevents further formation of toxic intermediates (ALA and PBG), reducing clinical signs of porphyria. Abbreviations: AGO, Argonaute 2; Dicer, ribonuclease enzyme; PACT, Protein activator of the interferon-induced protein kinase (PKR); RISC, RNA-induced silencing complex; TRBP, HIV-1 Trans-activation response (TAR) RNA-binding protein.

Givosiran was the second FDA-approved siRNA and brought two significant improvements compared to patisiran: the absence of a nanoparticle for delivery and a different route of administration [63]. First, the company identified a ligand capable of vectorizing siRNAs to hepatocytes without the LNP previously used in patisiran, illustrating their commitment to site-specific delivery [69]. Consequently, a novel hepatic-targeting strategy was developed by conjugating N-acetylgalactosamine (GalNAc) to the siRNA passenger strand (Figure 4). GalNAc specifically binds to the asialoglycoprotein receptor (ASGPR) on hepatocytes, facilitating efficient siRNA uptake and gene silencing without nanoparticle-based delivery systems (Figure 4C). Gene knockdown was observed in a dose-dependent manner [69,70,71].

Upon hepatocytes internalization, the siRNA duplex engages the Dicer/TRBP/PACT complex, which processes the duplex and loads the antisense strand into the RISC. Within RISC, the guide strand directs the complex to the complementary ALAS1 mRNA sequence, leading to its degradation through the RNAi pathway. This reduction in ALAS1 enzyme levels results in decreased synthesis of the neurotoxic heme intermediates δ-aminolevulinic acid (ALA) and porphobilinogen (PBG), addressing a central pathological mechanism in acute hepatic porphyria.

Second, a key advancement of the GalNAc-conjugated givosiran formulation is the transition from intravenous administration, which previously required medical supervision, to subcutaneous injection. This modification significantly enhances patient convenience and allows for a more accessible administration regimen at home [70,71].

In summary, the siRNAs that have received prior FDA approval illustrate significant advancements in RNAi biotechnology, particularly in targeted oligonucleotide delivery. This progress has been achieved by conjugating double-stranded RNAs with chemical compounds that improve vectorization efficacy. These developments have played a crucial role in the further advancement of C16 siRNAs. A comparative overview of these delivery strategies is presented in Table 1.

## 4. C16-siRNAs

The therapy for chronic-degenerative diseases of the central nervous system (CNS), whether stemming from genetic dysfunction or other origins, remains a significant challenge in the field of medicine [41,76]. Many of these diseases are characterized by the accumulation of pathological proteins that lead to neuronal death, as observed in conditions such as Parkinson’s and Alzheimer’s disease, with alpha-synuclein and Aβ peptides being notable examples, respectively [40,42,77]. In Parkinson’s disease (PD), despite the majority of cases being idiopathic, a percentage can be attributed to genetic mutations, including the A53T mutation, which involves the substitution of the amino acid alanine with threonine at position 53 [78]. This mutation promotes the formation of protein aggregates, resulting in neuronal damage. In genetic disorders that cause neurological impairments, such as de novo DEAF1 (deformed epidermal autoregulatory factor-1), specific mutations compromise the function of this protein, which serves as a zinc finger to regulate the activity of promoter regions, thereby adversely affecting the expression of various cerebral genes [79,80,81].

Given these examples, silencing pathogenic proteins involved in brain diseases through RNAi has emerged as a promising alternative. As mentioned, this approach became more tangible in 2018 when the FDA approved the first siRNA, patisiran. While siRNA therapies like patisiran and givosiran have demonstrated efficacy in treating liver diseases through vectorization molecules, targeting the brain poses a significant challenge, particularly in delivering oligonucleotides across the blood–brain barrier and effectively transfecting neuronal cells [82,83]. This limitation led to skepticism regarding the progress of gene therapy in the CNS, where many diseases manifest [84]. Thus, developing an effective strategy to overcome these obstacles is commendable [85,86,87].

The seminal study conducted by Brown et al., published in 2022, provided compelling evidence that the attachment of a hexadecyl lipid at the 2’ position of the ribose in siRNA molecules significantly enhances their biodistribution across various brain regions [72]. These meticulously engineered oligonucleotides, known as 2’-O-Hexadecyl (C16)-siRNA conjugates, are administered via the IT route, representing a novel strategy for delivering siRNA-based therapeutics for neurological disorders.

Conjugation of siRNA with a C16 (palmitic acid) chain markedly enhances its penetration and distribution within the CNS by modulating lipid–protein interactions, endocytic uptake, and parenchymal diffusion. The hydrophobic palmitate moiety increases siRNA affinity for cellular membranes and endogenous lipid carriers, including albumin and lipoproteins, which facilitate receptor-mediated transcytosis across endothelial and glial barriers. Chappell et al. (2020) demonstrated that C16-conjugated oligonucleotides exhibit high-affinity binding to plasma proteins, resulting in prolonged systemic circulation and controlled release into tissues, where albumin-mediated transport enables enhanced tissue permeability [73]. These interactions also favor engagement with scavenger receptors such as scavenger receptor B, type I (SR-BI) and low-density lipoprotein receptor (LDLR), as shown by Wolfrum et al. (2007), which mediate internalization via clathrin- and caveolae-dependent endocytosis [88]. Within the CNS, Chen et al. (2010) observed that lipidated siRNAs efficiently translocate into oligodendrocytes and neurons, displaying trafficking through early endosomes and recycling compartments prior to cytosolic release [89]. This process likely benefits from the palmitate anchor’s ability to transiently associate with lipid bilayers, promoting endosomal escape through localized membrane perturbation. Collectively, these studies indicate that C16 conjugation enhances CNS penetration by stabilizing siRNA–protein complexes in circulation, enabling receptor-mediated endocytosis at the blood–brain barrier, and facilitating intracellular trafficking routes that culminate in effective cytosolic delivery and sustained gene silencing within neural tissues [88,89].

Furthermore, C16-siRNAs demonstrate notable biodistribution in ocular and pulmonary tissues, thereby expanding the potential for addressing diseases affecting these important organs [72].

The research on C16 siRNAs began over a decade before the publication of the article [72]. In 2019, Alnylam Pharmaceuticals, Inc. was granted a patent titled “Extrahepatic Delivery” (WO 2019/217459 A1). The abstract of the patent states: ‘The invention relates to a method of gene silencing, comprising administering to a cell or a subject in need thereof a therapeutically effective amount of lipophilic moieties-conjugated double-stranded iRNAs at one or more internal positions on at least one strand, optionally via a linker or carrier.’ The term “therapeutically effective amount” holds particular importance in pharmacology, as it aligns with the widely recognized notion that a drug will produce the desired therapeutic effect when it reaches an adequate concentration in the target organ or biophase. This concept is thoroughly discussed in our pharmacology textbook, *Goodman & Gilman’s The Pharmacological Basis of Therapeutics*.

Vasant Jadhav and Martin Maier were the corresponding authors of the cited article [72] that warrants consideration for the cover of the *Nature Biotechnology* journal (https://www.nature.com/nbt/volumes/40/issues/10) (accessed on 15 December 2025). The proposed design features a detailed 3D model that illustrates the interaction between the C16-conjugated siRNA (depicted in orange), the RISC (shown in white), and the mRNA (represented in green) involved in the process of gene silencing. This model was constructed by Erin Dewalt from Alnylam Pharmaceuticals. At the top of the cover, the phrase “Extrahepatic Delivery of RNA Therapeutics” would prominently headline the visual representation [74].

The complete history of C16 development was told by the authors, who measured, across two decades, the time needed for scientific basic studies until testing the clinical potential of a siRNA-based drug [74]. Previous experiences with other siRNAs have made a significant contribution. The development of siRNAs and their efficient in vivo delivery to target cells is of paramount importance. For liver-targeted delivery, the strategy achieved was either by encapsulating siRNAs within LNPs or by directly conjugating stable siRNAs to a targeting ligand that includes GalNAc, recognized by the ASGPR present on liver hepatocytes, as previously discussed [63,68].

The authors referenced several studies investigating lipophilic compounds for conjugation with siRNAs, including cholesterol, aiming to achieve robust activity in biological systems, such as cell culture and animal model assays [90,91,92]. In this context, researchers have explored alternative molecules that could enhance the ability of siRNAs to cross the lipid bilayer of cells, thereby improving their lipophilicity and overall delivery efficiency. Importantly, these compounds are designed to be non-toxic to the organism. Vasant Jadhav and Martin Maier noted that the first successful outcome of an siRNA conjugated with a lipophilic compound was achieved in 2018 after IT injection in rats. Following this breakthrough, a series of assays were conducted to optimize the performance of this siRNA and obtain a product suitable for clinical application. This section will first provide an overview of siRNA itself, followed by a discussion of the experimental results demonstrating its enhanced distribution, particularly within the brain [72].

### Innovations in the Design of Brain-Delivered C16-siRNAs: A Functional Perspective

**Figure 5:**
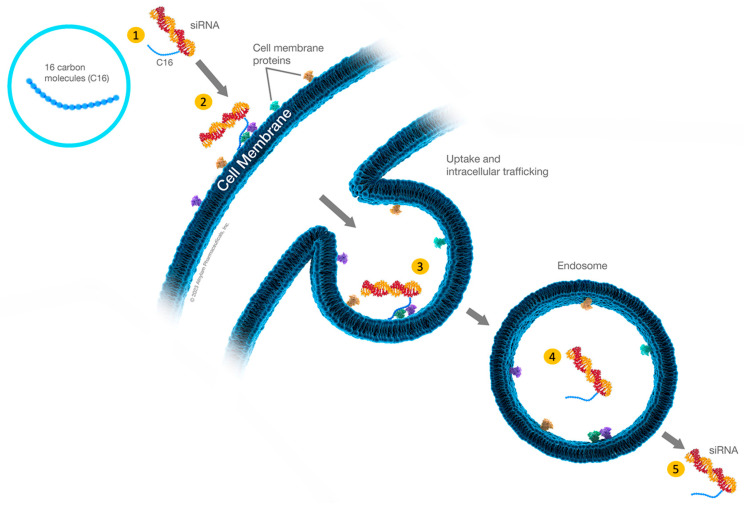
The role of C16 molecules in siRNA internalization across lipidic cell membranes. This illustration depicts the mechanism of internalization for C16-modified siRNA molecules into cells. (**1**) The 2’-O-hexadecyl (C16) modification is represented as a blue tail, signifying a short fatty acid chain that enhances the siRNA’s lipophilicity. (**2**) The C16 tail interacts with cellular membranes and membrane proteins, facilitating initial contact. (**3**,**4**) Subsequently, siRNA is internalized into endosomes. (**5**) Endosomal escape enables the siRNA to exit the endosomal compartment and initiate RNAi-mediated gene silencing. Courtesy of Alnylam Pharmaceuticals Inc. Modified from Anlylam, Delivery Platforms—C16 Conjugates (2023) [75]. Abbreviations: C16, 16-carbon-length molecule; siRNA, small interfering RNA.

C16 conjugates are characterized by the 2’-O-hexadecyl (C16) modification, which involves the covalent attachment of a short fatty acid chain to the siRNA molecule. This modification is illustrated in Figure 5 as a blue tail, identified as number 1 within the yellow-filled circle. The hydrophobic nature of this modification significantly enhances the lipophilicity of the siRNA, thereby improving its interaction with cellular membranes and associated membrane proteins. This interaction is illustrated by the blue C16 tail, which is connected to schematic representations of membrane proteins (number 2). Such interactions are crucial for the effective uptake of siRNA across diverse cell types, including those found in the CNS, as well as in lung and ocular tissues. The diagram also illustrates the mechanism of cellular uptake, which involves the internalization of siRNA into endosomes (numbers 3 and 4). Then occurs the endosomal escape, which is essential for allowing siRNA to exit the endosome and freely access the cytosol (number 5), where it can initiate its silencing effects, as previously described.

**Figure 6:**
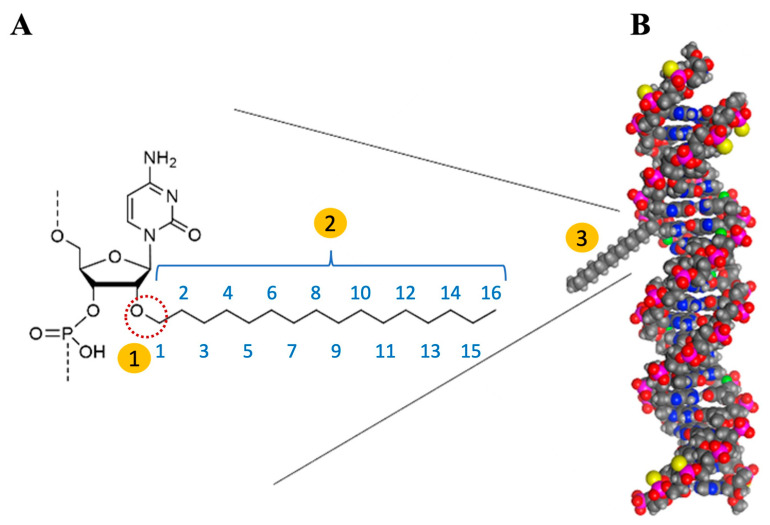
Schematic representation of a lipophilic-siRNA conjugate. This figure presents a chemical and spatial modeling approach illustrating the interaction between C16 and siRNA. (**1**) On the left (**A**), the oxygen atom at the 2′ position of the adenine ribose in the sense strand of siRNA interacts with the 16-carbon chain (2′-O-C16), indicated within a dashed red circle. (**2**) The central section displays the C16 chain, comprising 16 carbon atoms. (**3**) On the right (**B**), a spatial representation of siRNA is shown, with the C16 molecule depicted as a protruding gray structure. Modified from Vasant Jadhav and Martin Maier (2022) [74].

In Figure 6, we depict the interaction between C16 and siRNA from a chemical perspective and through spatial modeling, as outlined by Vasant Jadhav and Martin Maier [74]. The schematic representation illustrates the interaction between the oxygen atom located at the 2′ position of the adenine ribose in the sense strand of siRNA and the 16-carbon chain (2′-O-C16). This interaction is highlighted on the left by a dashed red circle and is labeled as number 1 within a yellow-filled circle. In the middle, the C16 chain, which consists of 16 carbon atoms, is identified as number 2. On the right, the figure presents a spatial representation of the siRNA, where the C16 molecule is depicted as a protruding gray structure (labeled as number 3). It is crucial to acknowledge that the lipophilic C16 molecule resembles a tail, functioning effectively as an anchor with significant spatial implications. While its size is relatively modest compared to the overall siRNA molecule, Brown et al. (2022) [72] undertook a thorough investigation of the potential effects of C16 incorporation and determined the optimal positioning for its integration, as detailed below. The spatial conformation of siRNA is pivotal for its recognition by the RISC and the subsequent initiation of RNAi-mediated gene silencing.

The authors conducted a series of experimental tests to optimize the design of C16-siRNAs, beginning with the most critical component: the lipophilic moiety. The C16, a relatively large molecule of 16 carbon atoms, was conjugated to a small double-stranded RNA with 21 nucleotides (siRNA moiety) to enhance biodistribution across CNS regions. This modification was meticulously considered to ensure that it did not interfere with silencing efficacy, which depends on the incorporation and activation of the RISC, as previously outlined.

To achieve the 16 carbon chain length, this study investigated the in vivo efficacy of siRNAs targeting the enzyme copper-zinc superoxide dismutase 1 (SOD1), composed of 10, 12, 14, 16, and 18 carbon atoms [72]. RNAi effects were assessed in the rat’s brain and spinal cord tissues two weeks after IT administration of 0.9 mg of siRNA (n = 4 per group). Furthermore, the research explored the optimal positioning for the attachment of the C16 tail by evaluating the silencing efficiency of siRNA attachments at various nucleotide positions on the sense (1–21) and antisense (2–23) strands in cell culture. In rat tissues, the evaluated sense strands included nucleotides 1, 2, 5, 6, 7, 10, 11, 16, 17, and 21, while the antisense strands comprised nucleotides 5, 15, and 16, with assessments conducted on day 28. The most effective RNAi silencing was observed at nucleotide number 6 of the sense strand. At this point, the research has successfully identified the optimal lipophilic chain length of 16 carbon atoms, as well as the advantageous attachment position at the 6th nucleotide of the sense strand of the siRNAs [72].

**Figure 7:**
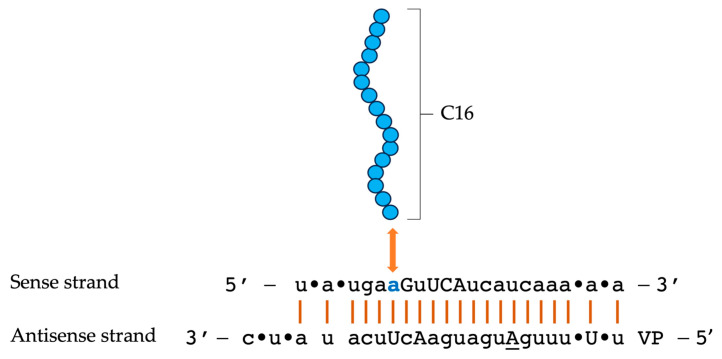
Structure of Human APP-Targeting siRNA XVIII. This illustration depicts the C16-siRNA structure, as referenced in Brown et al. (2022) [72]. The key components include: (i) the sixteen carbon atoms of the lipophilic moiety (C16), represented by blue-filled circles; (ii) a double-headed orange arrow indicating the conjugation of the C16 ligand to the 2’ position of the pentose in the adenine nucleotide (colored blue); (iii) upper-case and lower-case letters denoting the 2′-deoxy-2′-fluoro (2′-F) and 2′-O-methyl (2′-OMe) ribosugar modifications, respectively; (iv) vertical orange lines connecting the sense and antisense strands, illustrating Watson and Crick hydrogen bonds between complementary nucleotides; (v) underlined uppercase letter A in the antisense strand indicates a modification of glycol nucleic acid (GNA); (vi) the symbol (•) indicating phosphorothioate (PS) linkages; and (vii) “VP,” which refers to the incorporation of 5’-(E)-vinylphosphonate. Abbreviations: C16, 16-carbon-length molecule; VP, vinylphosphonate.

The double-stranded C16-siRNA structure, composed of 21 nucleotides, was designated as compound number XVIII, as outlined in Supplementary Table 1 of the original article [72]. Figure 7 illustrates the key components, highlighting the central feature: the sixteen carbon atoms of the lipophilic moiety, referred to as C16, which are represented by 16 blue-filled circles. A double-headed orange arrow indicates the conjugation of the 2′-O-C16 ligand to the sixth nucleotide from the 5’ end of the sense strand, N6, which is shown as the adenine nucleotide (also depicted in blue). Upper-case and lower-case letters in the diagram signify the modifications of the ribosugar: upper-case for 2′-deoxy-2′-fluoro (2′-F) and lower-case for 2′-O-methyl (2′-OMe). Additionally, the underlined uppercase letters, such as the letter “A” found in the antisense strand, signify a specific modification of glycol nucleic acid (GNA). The symbol (•) denotes phosphorothioate (PS) linkages, while “VP” indicates the presence of 5’-(E)-vinylphosphonate [72].

Important additional enhancements aimed to improve siRNA efficacy included a series of chemical modifications that were gradually integrated into the original siRNAs developed by the manufacturer, such as patisiran and givosiran [63,68]. Notably, one of the primary modifications involved substituting certain phosphodiester linkages with phosphorothioate (PS) linkages. This alteration was specifically aimed at decreasing the susceptibility of siRNA to enzymatic degradation, particularly by ribonucleases, thereby improving the overall stability of the siRNA molecules. These structural changes are critical for increasing the therapeutic potential of siRNAs by ensuring longer-lasting effects in biological systems [48,93].

Incorporating 2′-deoxy-2′-fluoro and 2′-O-methyl modifications in C16-siRNAs is a feature previously utilized in other siRNAs developed by Alnylam, resulting in enhanced potency, as reported in the literature [94,95,96,97,98,99]. Additionally, the C16-siRNA contains GNA in the antisense seed region, which spans from the second to the eighth base from the 5’ end, contributing to increased specificity in vivo [100,101]. Furthermore, the authors incorporated a vinylphosphonate at the 5’ end of the antisense strand [72]. This stable phosphate mimic enhances the efficacy of siRNAs in vivo by improving loading onto the RISC, ultimately increasing the potency of RNAi in cells [102,103]. SiRNAs engineered with both C16 and the 5’-vinylphosphonate modifications demonstrated increased RNAi activity compared to the individual components, achieving 75% and 95% silencing of SOD1 rat mRNA in the brain and spinal cord on day 28, respectively, following IT administration of 0.9 mg [72].

**Figure 8:**
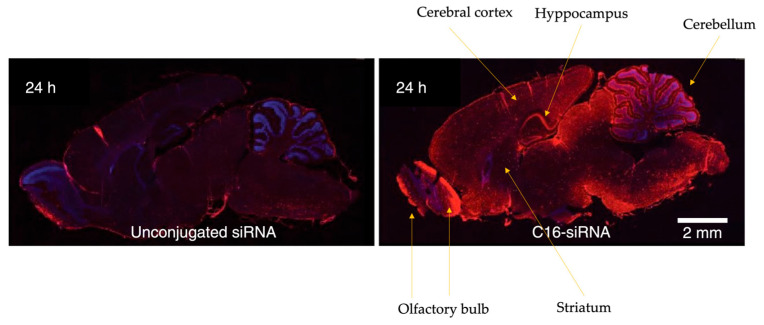
Biodistribution of C16-siRNAs across various brain regions after IT injection in rats. Immunohistochemistry (IHC) was performed using an anti-siRNA rabbit polyclonal antibody, which was detected with an anti-rabbit HRP secondary antibody. C16-siRNAs (**right**) demonstrated substantial penetration compared to unconjugated siRNAs (**left**), effectively reaching the cortex and hippocampus. Additionally, signals were detected in the olfactory bulb and brainstem. Modified from Brown et al. (2022) [72]. Abbreviations: siRNA, small interfering RNA; C16-siRNA, siRNA conjugated with C16, a 16-carbon-length molecule.

An elegantly detailed image of the work was produced using immunohistochemistry (IHC) with an in-house anti-siRNA rabbit polyclonal antibody, which was subsequently detected with an anti-rabbit HRP secondary antibody [72]. This analysis revealed a notable penetration of siRNAs conjugated with C16 on the right, in contrast to the unconjugated ones on the left (Figure 8). C6-siRNAs demonstrated a notable biodistribution throughout the rat brain following intrathecal injection, effectively reaching the cortex and hippocampus—regions critically involved in cognition and memory, which are negatively impacted in AD, the target condition for the current C16-siRNA therapeutics. Additionally, the signal was observed in the olfactory bulb and brainstem. Interestingly, C16-siRNAs exhibited limited penetration into the striatum, an area of considerable relevance in other neurodegenerative disorders, such as PD.

During the progression of neurodegenerative disorders such as AD, various cell types are involved, including neurons and glial cells associated with neuroinflammation, particularly astrocytes and microglia [104,105]. Thus, the cellular uptake of siRNAs across these different cell types is crucial for the therapeutic outcome. To investigate this aspect, the authors conducted IHC assays using siRNAs targeted to mRNAs uniquely expressed in the studied cell types. Neurons, astrocytes, microglia, oligodendrocytes, and perivascular/endothelial macrophages were identified by immunodetection of MAP2, GFAP, Iba1, MBP, and PECAM1, respectively. The results demonstrated successful uptake of the siRNAs in neurons, astrocytes, and microglia. In contrast, no signals were detected in oligodendrocytes [72].

**Figure 9:**
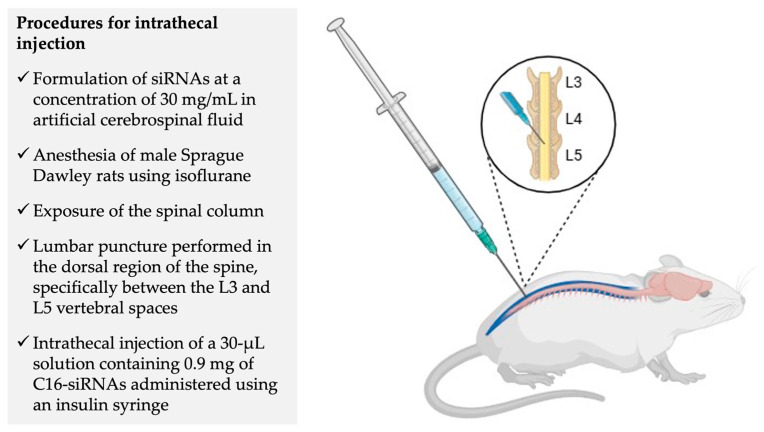
Intrathecal injection of C16-siRNAs. Intrathecal (IT) injections were performed in male Sprague Dawley rats using C16-siRNAs diluted in artificial cerebrospinal fluid at 30 mg/mL concentration. A total volume of 30 μL was administered, resulting in a dose of 0.9 mg per animal. The IT injection was executed by puncturing the lumbar region of the spinal cord between the L3 and L5 vertebrae. Abbreviations: siRNA, small interfering RNA; L3, L4, and L5 represent the lumbar vertebrae regions.

It is important to clarify the route of administration for this innovative siRNA delivered to the CNS: IT injection. IT injection was performed in male Sprague Dawley rats. The C16-siRNAs were initially diluted in artificial cerebrospinal fluid (aCSF) at a concentration of 30 mg/mL, and a volume of 30 μL was administered. Consequently, each animal received an administered dose of 0.9 mg. The IT injection was executed by puncturing the lumbar region of the spinal cord between the L3 and L5 vertebrae (Figure 9). Notably, there was cranial distribution of the siRNAs, presumably via the cerebrospinal fluid (CSF), from the lumbar injection site to other spinal regions and brain areas, as evidenced by qPCR results. Three spinal cord regions were assessed: lumbar, thoracic, and cervical, in addition to the cerebellum and brainstem. Gene expression assays via RT-qPCR were consistent with the biodistribution results obtained from IHC in the aforementioned areas. A marked silencing of the targets under investigation was observed following IT injection of 0.9 mg siRNA in neurons (MAP2 C16-siRNA at 28 days), astrocytes (GFAP C16-siRNA at 14 days), and microglia (Iba1 C16-siRNA at 32 days) (n = 3–6 animals per group) [72].

The visual analysis of the data indicates a greater intensity of gene silencing in the spinal cord segments, particularly in regions closer to the IT site, compared to the cerebellum and brainstem, specifically for GFAP, Iba1, and MAP2. The authors also described the effects of gene silencing, with GFAP, Iba1, and MAP2 levels reduced to less than 50% in all spinal cord segments. In the cerebellum, reductions below 50% were observed for GFAP and MAP2, indicating effects on astrocytes and neurons, but not on microglia. Similar reductions were noted in the brainstem for GFAP and Iba1, though not for MAP2. As statistical analyses of the experiments were not provided, it is prudent to consider that the observed differences may not have reached statistical significance. However, it can be inferred that the efficacy of gene silencing following IT injection in the L3–L5 lumbar region was influenced by the distance from the injection site and the specific neuronal or glial cell type present in the spinal cord, cerebellum, and brainstem. As anticipated, silencing effects were minimal in oligodendrocytes and vascular/perivascular macrophages, suggesting that other neuropathologies involving these specific cell types may not benefit from treatment with C16-siRNAs [72].

This anatomical aspect represents a limitation of the IT administration route in the lumbar region, particularly considering the necessity for siRNA to reach more cranial areas of the CNS, such as the cerebral cortex and hippocampus, which are affected in AD. However, the targets examined (GFAP, Iba1, and MAP2) were utilized more to characterize the penetration of siRNAs into different cell types rather than to present a pattern of physiological gene expression within the brain. In this context, the authors aim to conduct further tests utilizing the mRNA of the enzyme SOD1 as the target [72]. SOD1 is an intracellular enzyme with antioxidant properties, thereby regulating baseline levels of oxidative stress in healthy individuals, while also playing a role in mechanisms underlying various neurodegenerative diseases, including amyotrophic lateral sclerosis (ALS) and PD [106]. The genetic structure of SOD1 and its gene expression regulation are well characterized, making it a widely expressed gene within the nervous system and suitable for assessing the efficacy of silencing systems such as those described in this study [107,108].

The study conducted two significant pharmacological analyses: a dose–response curve and a temporal analysis regarding the duration of siRNA silencing, initiated after a single IT administration of 0.9 mg [72]. The siRNAs reduced the expression of SOD1 mRNA to below 50% for all three tested doses (0.07 mg, 0.3 mg, and 0.9 mg) at day 28, across the thoracic spinal cord, cerebellum, and frontal cortex. Notably, only the experimental group that received the lowest dose of 0.07 mg exhibited a minimal reduction in the frontal cortex [72].

The silencing effect was found to be dose-dependent, with the highest dose of 0.9 mg reducing SOD1 mRNA to 25% of the control value in all three regions assessed. This dose also produced a durable effect across various brain regions, with a silencing effect exceeding 50% that persisted for over three months in most regions analyzed, including all spinal cord segments. The silencing effects followed an expected gradient, being most pronounced near the injection site (lumbar), followed by thoracic and cervical regions. Additionally, effects were observed in the cerebellum, frontal cortex, temporal cortex, and hippocampus [72].

In both the spinal cord and frontal cortex, the RNAi effect remained above 50% after five months post-IT injection. The authors also tested the administration of a reduced dose of one-third of the original concentration (0.3 mg), given monthly for five months, which yielded positive results [72].

Assays conducted with NHPs confirmed the findings obtained from rat studies. In this research phase, cynomolgus monkeys were administered 60 mg of IT siRNAs. The results indicated that the efficacy of siRNAs featuring both the C16 tail and VP modifications was higher than that of the isolated use of these modifications in most brain regions. Similar to the rat studies, NHPs exhibited limited siRNA penetration in the striatum. An important finding in rats and NHP was the absence of RNAi effects on organs outside the CNS, liver, and kidney. This result indicates that the C16-siRNAs administered via the IT route maintain a target-specific distribution. Notably, the siRNAs reduced the CSF levels of sAPPα and sAPPβ to below 25% for up to two months, and subsequently to below 50% for five months [72].

The authors subsequently conducted studies using the CVN mouse model of AD, administering siRNA via the intracerebroventricular (ICV) route at a dose of 60 μg. The siRNA treatment reduced APP expression at 30 days post-administration, affecting both mRNA and peptide levels. Notably, the effect on APP mRNA in animals treated with 120 μg persisted for 60 days in the ventral cortex and was also observed in other brain regions over extended time points. Additionally, behavioral assessments indicated changes in total distance traveled and rearing frequency during the open-field test in the siRNA-treated animals, as discussed in a further topic of this review [72].

Based on the positive pharmacological results from preclinical testing of C16-siRNAs in rodents and NHPs, which demonstrated their potential for durably lowering amyloid precursor mRNAs and protein levels, the compound has progressed to a Phase 1 clinical trial involving patients with early-onset Alzheimer’s disease (EOAD).

## 5. Phase 1 Clinical Trial—NCT05231785: Experimental Design and Preliminary Results

The Phase 1 clinical study (ALN-APP-001, mivelsiran; NCT05231785) is a multi-center, randomized, double-blind, placebo-controlled trial designed to evaluate the safety, tolerability, pharmacokinetics (PK), and pharmacodynamics of intrathecally administered ALN-APP in patients with EOAD. The trial is structured in two parts: Part A: Single ascending dose (SAD); Part B: Multiple ascending dose (MAD) [109].

ALN-APP utilizes Alnylam’s proprietary C16-siRNA conjugate technology to facilitate enhanced delivery into CNS cells via IT administration. The prolonged pharmacodynamic effect suggests sustained CNS exposure and long tissue half-life, although complete PK profiles are still under analysis. The IT route allows direct access to the CSF, maximizing CNS bioavailability while minimizing systemic exposure.

Eligible participants were adults with symptom onset before age 65 diagnosed with EOAD and a Mini-Mental State Examination (MMSE) score greater than 20 [110]. All participants had confirmed amyloid pathology through CSF analysis or positron emission tomography (PET) imaging. They were at the stage of mild cognitive impairment (MCI) or mild dementia, representing early-stage AD in a young-onset population. Unlike traditional Phase 1 trials conducted in healthy individuals, this study was designed to evaluate safety and preliminary efficacy in patients who may benefit from the reduction or suppression of amyloid production [111].

In the initial patient cohort, 12 participants were enrolled and randomized in a 2:1 ratio to receive either ALN-APP or placebo within the 25 mg and 75 mg siRNA dose groups. ALN-APP demonstrated robust target engagement following a single IT administration. Dose-dependent reductions in CSF levels of sAPPα and sAPPβ were observed on day 15 post-administration. Mean reductions from baseline were 55% for sAPPα and 69% for sAPPβ, with maximal reductions of 71% and 83%, respectively, observed in the 75 mg cohort (n = 4) [110].

ALN-APP also demonstrated a favorable safety profile in this initial cohort. All reported adverse events (AEs) were mild to moderate in severity. The most commonly observed AEs included back pain, headache, post-lumbar puncture syndrome, and syncope, each occurring in 2 participants (17%). Additional AEs were reported in 1 participant each (8%) and included constipation, hemorrhoids, injection site pain, procedural pain, elevated alanine aminotransferase (ALT), amyloid-related imaging abnormalities with microhemorrhages and hemosiderin deposits (ARIA-H), presyncope, and vomiting. No serious AEs or dose-limiting toxicities were observed. Moreover, no safety signals were identified based on routine CSF biomarkers or exploratory markers such as neurofilament light chain (NfL) [110].

The clinical trial data discussed herein represent preliminary results, and the publication by Cohen et al. (2023) does not include statistical treatment of these findings [110]. Caution is therefore warranted when interpreting these outcomes. The study presents results from a limited sample size (n = 4 individuals per group), including placebo, 25 mg, and 75 mg treatment arms. Consequently, the observed changes in β-amyloid α and β levels and the reported adverse effects should be considered exploratory. Further analyses with larger cohorts and appropriate statistical validation are required to confirm these preliminary observations.

In a new cohort study involving 36 patients with EOAD (mean age 60.9 years; mean MMSE score 24.6), participants were assigned to escalating dose cohorts (25, 35, 50, and 75 mg) and evaluated over a minimum follow-up period of six months. Extended monitoring continued for up to 12 months to assess the durability of treatment effects [112]. CSF samples were collected on Day 15 and at Months 1, 2, 3, 4, 5, and 6 to analyze APP and Aβ biomarkers [111].

AEs were common; however, most were mild to moderate in severity and not classified as serious. The most frequently reported AE was post–lumbar puncture headache, observed in 41.7% of participants. This event was more prevalent in the lower dose groups, occurring in 75% of participants in the 50 mg cohort, 50% in both the 25 and 35 mg cohorts, and only 14.3% in the 75 mg cohort [112].

Only three participants experienced AEs that were considered possibly related to the investigational drug. One individual in the 50 mg group reported mild headache and nausea, both deemed related to IT administration as well as to the study drug. Two participants in the 75 mg group experienced moderate AEs, including headache, neck pain, vomiting, and lymphocytopenia. The first three events were also attributed to the lumbar puncture procedure. A serious and fatal AE of acute pancreatitis was reported in the 75 mg group on day 277 following administration of a single dose; however, this event was assessed as unrelated to ALN-APP mivelsiran [112].

Regarding pharmacodynamics, a dose-dependent and sustained reduction in the levels of sAPPα and sAPPβ was observed in the CSF. In the 25 mg cohort, reductions were modest and transient. In contrast, 35 mg and above doses resulted in marked and sustained decreases. The 75 mg dose elicited the most pronounced effect, with mean reductions of 61.3% in sAPPα and 73.5% in sAPPβ observed one month post-administration. These reductions were maintained through six months in all cohorts receiving ≥ 35 mg, and persisted for up to 12 months in the 50 and 75 mg groups [112].

In addition, the effects of mivelsiran on Aβ peptides were assessed. Reductions in CSF Aβ42 and Aβ40 levels were noted in cohorts receiving 35 mg or higher, with the magnitude of reduction increasing with dose escalation. Once again, the 75 mg dose was the most effective, achieving mean reductions of 49.3% for Aβ42 and 66.5% for Aβ40 at one month. These effects were sustained for at least six months in the 35 mg, 50 mg, and 75 mg groups, suggesting effective inhibition of the amyloidogenic cascade in response to APP gene silencing [112].

These findings support the hypothesis that ALN-APP may suppress the production of all APP-derived cleavage products, both intracellular and extracellular, thereby potentially addressing multiple pathological pathways involved in AD and cerebral amyloid angiopathy (CAA).

The pharmacodynamic effects’ consistency and a favorable safety profile support the continued clinical development of ALN-APP as a potential disease-modifying therapy for AD and other conditions associated with Aβ deposition, such as cerebral amyloid angiopathy [112].

## 6. A Critical Analysis of Disease Phenotypes and Animal Models Used in Preclinical Studies

Patients recruited for the Phase 1 clinical trial (NCT05231785) present with a condition known as EOAD. This form of the disease typically manifests before the age of 65 and is characterized by a more aggressive progression, affecting approximately 5–10% of all AD patients [113]. Memory impairments in EOAD may be accompanied by various cognitive deficits across multiple domains, including visuospatial, language, and executive functions. Notably, some patients exhibit less pronounced amnesic symptoms, with episodic memory relatively preserved; instead, they may present with other focal cortical manifestations, such as language impairment, visuospatial deficits, executive dysfunction, and motor impairments [114,115,116].

Furthermore, only individuals diagnosed with MCI or mild dementia attributable to EOAD were included in the study. Participants’ cognitive status was assessed using the Clinical Dementia Rating (CDR) global score, with acceptable scores being either 0.5 or 1.0 [117]. Additionally, participants were required to achieve an MMSE score greater than 20 [118,119].

The scientific community eagerly awaits reports on the clinical benefits of siRNA ALN-APP for patients with EOAD, particularly concerning cognition and memory, which are key phenotypes of AD. However, the ongoing Phase 1 study of siRNA ALN-APP (NCT05231785) is expected to conclude in March 2029 with a primary focus on endpoints that do not include mental outcomes. Specifically, the study focuses on safety, tolerability, and the effects on APP, among other parameters. The trial evaluates the incidence of adverse events, overall survival, and pharmacodynamic responses associated with APP levels, which are measured in CSF, plasma, and urine. Despite this, our research group reached out to the coordinator of clinical studies in September 2025 to inquire about the availability of data on cognitive or memory phenotypes, but we were informed that no preliminary findings in this regard have been reported.

A potential strategy for elucidating the effects of siRNA on amyloid deposition and brain function may involve using animal models in preclinical testing. Transgenic animal models of AD have enabled investigations into the neurobiology of this condition and the responses to various interventions. These models play a crucial role in scientific research to identify potential therapeutic targets and in the preclinical studies that precede human testing [120,121]. However, it is widely acknowledged that animal models provide only partial representations of human disease, given the differences in neuropathological aspects and the complex clinical manifestations, such as advanced cognitive functions and memory, which can only be partially evaluated through animal experimentation. Consequently, significant limitations are associated with using these models to translate laboratory findings into clinically relevant information. Nonetheless, they remain indispensable experimental systems for enhancing our understanding of AD and facilitating preclinical testing [122,123,124].

One approach employed in generating transgenic animal models for AD involves introducing specific genetic mutations into mice. These mutations have been described in the same condition found in patients recruited for Phase 1 clinical trials, EOAD [125,126,127]. Pathogenic variants are frequently found in genes encoding APP, Presenilin 1 (PSEN1), and Presenilin 2 (PSEN2) [116,128,129]. These transgenic models are invaluable for investigating disease progression mechanisms and testing potential therapeutic interventions.

In summary, a significant advantage of the transgenic mice used in preclinical testing is their incorporation of pathogenic mutations and some phenotypes similar to those observed in patients with EOAD participating in the C16 ALN-APP Phase 1 clinical trial.

Although there are inherent limitations in using animal models to replicate human disease accurately, we assert a satisfactory correspondence between the chosen animal models and the human condition under investigation, as outlined in the following discussion.

Two transgenic mouse lines were utilized in the testing of siRNA C16-ALN APP. The first line, evaluated in the preclinical efficacy trials reported by Brown et al. (2022), consisted of homozygous Tg-hAPPSwDl/mNos2^−/−^ mice, referred to as CVN mice (cerebrovascular amyloid Nos2^−/−^ or CVN-AD), aged between 6 and 12 months [72]. These transgenic mice express human pathogenic variants of the APP, including the Swedish K670N/M671L, Dutch E693Q, and Iowa D694N mutations, crossed with a Nos2^−/−^ background [130,131]. Research demonstrated that this animal model develops a series of pathological events leading to neurodegeneration. This progression begins with the pathologically hyperphosphorylated tau in the perforant pathway at 6 weeks of age, followed by the formation of insoluble tau aggregates. Additionally, early appearances of Aβ peptides are observed as perivascular deposits around blood vessels in brain regions known to be vulnerable to AD. Ultimately, this sequence of events results in neuronal damage and significant loss within specific vulnerable neuronal populations in these affected regions.

Using the same model, Brown et al. (2022) examined the expression of amyloid beta and Iba1 (a marker for microgliosis) through IHC in the brains of mice treated with C16-ALN APP via ICV administration at 9 months of age [72]. Immunostaining for Aβ 40 in the cortex and hippocampus was slightly reduced in the treated groups; however, this difference was not statistically significant. Regarding neuroinflammation, treated mice exhibited a significantly lower increase in Iba1 immunostaining in both the cortex and hippocampus between 6 and 9 months of age compared to controls, indicating a reduction in microgliosis.

Furthermore, the study assessed behavioral changes in the treated animals using an open field apparatus that measured distance traveled, number of vertical rearings, and average velocity. Treatment with siRNAs demonstrated positive effects on two of these parameters. Treated CVN mice displayed a reduced distance traveled, suggesting that the treatment mitigated hyperactivity, as well as a lower frequency of rearing compared to untreated CVN animals [72].

A recent study presented at the Alzheimer’s Association International Conference (AAIC) 2025 utilized another transgenic mouse model to investigate the effects of ICV administration of ALN-APP siRNAs on AD pathology and associated behavioral alterations [132]. The mice employed in this study coexpress five mutations associated with Familial Alzheimer’s Disease (FAD), referred to as 5xFAD mice [133]. This model is characterized by the overexpression of a mutant human APP containing the Swedish (K670N, M671L), Florida (I716V), and London (V717I) FAD mutations. Additionally, the model incorporates the human PS1 gene, which harbors two further FAD mutations, M146L and L286V. Notably, the overexpression of these transgenes in the brain is regulated by neural-specific elements derived from the mouse Thy1 promoter. The widely utilized 5xFAD model exhibits pronounced amyloid plaque deposition, reactive gliosis, behavioral changes, and other pathological features characteristic of AD. Nevertheless, as with other neurodegenerative disease models, it has limitations that may hinder its capacity to fully represent the complex spectrum of the disease, as discussed in other reviews [134,135,136,137].

Initially, the authors employed the Meso Scale Discovery (MSD) immunoassay to quantify Aβ40 and Aβ42 levels in cortical samples [132]. All early intervention doses—transient, moderate, and robust—significantly decreased both Aβ40 and Aβ42 concentrations. Similarly, treatments administered in the late intervention group also led to a notable reduction in Aβ40 and Aβ42 levels. In the early intervention group, the authors observed a decrease in plasma NfL and a significant reduction in glial fibrillary acidic protein (GFAP) as measured by IHC for both moderate and robust treatments. The late intervention group also exhibited a reduced plasma NfL and a significant decrease in GFAP levels. The authors further investigated the potential impact of transient, moderate, or robust treatments on behavior utilizing the elevated plus maze (EPM), a widely used model for assessing anxiety-related behaviors and evaluating pharmacological interventions. The apparatus comprises an elevated maze with two open arms and two enclosed arms; animals typically exhibit fewer entries into the open arms, which represent a more aversive environment, and spend less time there compared to the closed arms. Anxiolytic drugs are known to increase both the number of entries and the proportion of time spent in the open arms [138,139].

Behavioral alterations in 5xFAD mice have been assessed using various tests, including the EPM, open field, and contextual fear conditioning [121,135,136]. A significant finding in 5xFAD mice is the abnormal reduction in anxiety-like behavior as evidenced by increased time spent in the open arms of EPM [135,136,140,141]. In the cited study, Taillie et al. (2025) found that treatment with C16 ALN-APP siRNAs resulted in a dose-dependent decrease in the time spent in the open arms when comparing the early intervention protocol to the untreated 5xFAD control group [132]. The highest siRNA dose of 300 µg administered via ICV at 3 and 6 months, referred to as robust intervention, produced the most pronounced effects.

Notably, the exploratory behavior of 5xFAD mice receiving this robust intervention was comparable to that of healthy control animals at 8 and 12 months. In contrast, the late intervention protocol (300 µg ICV at 8 months of age), despite eliciting reductions in Aβ40, Aβ42, NfL, and GFAP levels, did not mitigate the abnormal behavior characterized by increased time spent in the open arms. This finding underscores the critical importance of initiating siRNA treatments at the earliest pathological stages of the disease.

## 7. Analysis of Dose-Escalation Testing on Silencing Effects and Tolerability in Clinical and Preclinical Studies

Determining the maximum tolerated dose (MTD) and the associated risk-benefit ratio are critical considerations in developing new therapeutic drugs. This complexity is particularly pronounced in RNAi-based therapies, which are influenced by several intrinsic factors unique to this class of medications. The nature of the treatment agent—specifically, nucleic acids—and factors such as cost and pharmacodynamics, play significant roles in this context. For instance, the dosing of small interfering RNA (siRNA) must be carefully regulated to achieve the desired silencing effects, contingent upon cellular mechanisms’ functionality, notably the RISC [48]. Consequently, specific RNAi-based therapies, such as patisiran and givosiran, have received FDA approval without having an established MTD. This underscores the nuanced considerations involved in the therapeutic usage and evaluation of RNAi treatments.

A first example refers to the clinical investigation of patisiran, in which small interfering RNA (siRNA) doses were evaluated in patients diagnosed with transthyretin familial amyloidotic polyneuropathy (TTR-FAP) [142]. This Phase II dose-escalation study involved 26 patients who received two intravenous (IV) infusions of varying doses (0.01, 0.05, 0.15, and 0.3 mg/kg) administered every four weeks, and a regimen of 0.3 mg/kg every three weeks. The results demonstrated a dose-dependent reduction in serum transthyretin (TTR) levels, achieving a maximum knockdown of 96% with the 0.3 mg/kg dose administered every three weeks. Dose-limiting toxicities were defined by several criteria, including any life-threatening toxicity, elevated ALT and aspartate aminotransferase levels ≥ 5 times the upper limit of normal, a total bilirubin level exceeding 2.0 mg/dL, infusion reactions necessitating hospitalization, and any other toxicity deemed by the Safety Review Committee to preclude the administration of subsequent doses. The most prevalent treatment-emergent adverse event associated with the study drug was a mild-to-moderate infusion-related reaction, which occurred in 3 out of 29 patients overall (10.3%), predominantly within the 0.3 mg/kg dose group administered every four weeks. Importantly, none of these treatment-emergent AEs led to discontinuation of the treatment regimen. Throughout the study, there were no reports of dose-limiting toxicities or deaths attributable to treatment-emergent adverse events. Most recorded treatment-emergent AEs were classified as mild or moderate in intensity [142].

A second example of toxicity analysis pertains to another formulation for subcutaneous injection of TTR siRNAs, named ALN-TTRsc02. This GalNAc-conjugated drug is designed for the treatment of hereditary ATTR (hATTR) amyloidosis [143]. The Phase I trial (NCT02797847) assessed the safety and tolerability of ALN-TTRsc02 in healthy volunteers, who were administered either the drug (n = 60) or a placebo (n = 20). All tested doses—5, 25, 50, 100, 200, and 300 mg—demonstrated acceptable tolerability profiles. Based on modeling data and the duration of effects observed at clinically relevant doses, the authors proposed that ALN-TTRsc02 has the potential to reduce hepatic TTR production [143].

Finally, a third example of toxicity analysis involves givosiran, a siRNA utilized to treat porphyria [144]. The clinical trial was structured into three parts: Parts A and B included patients who had not experienced porphyria attacks in the previous six months (n = 23). Part C focused on patients who had experienced porphyria attacks during the same timeframe or were undergoing a heme injection regimen (n = 13 for givosiran; n = 4 for placebo); these participants were required to discontinue hemin treatment during both the hospitalization and intervention periods. In Part A, patients were randomized in a 3:1 ratio to receive a single subcutaneous injection of either givosiran or placebo. The ascending doses evaluated included 0.035, 0.10, 0.35, 1.0, and 2.5 mg/kg. Part B also randomized patients in a 3:1 ratio to receive monthly subcutaneous injections of either 0.35 or 1.0 mg/kg. Patients from Part C, who were experiencing recurrent attacks of acute intermittent porphyria (AIP), received subcutaneous administrations of givosiran at higher doses of 2.5 or 5.0 mg/kg, either monthly (totaling four injections) or at three-month intervals (totaling two injections) over a period of 12 weeks.

From a safety perspective, the authors observed no significant difference in the frequency of AEs reported between the givosiran and placebo treatment groups. Notably, adverse effects were reported in 91% (30/33) of patients receiving givosiran compared to 100% (10/10) in the placebo group. The most frequently reported AEs during the trial included nasopharyngitis, abdominal pain, nausea, and diarrhea [144].

In conclusion, the definition of the MTD has not been rigorously established during the development of small siRNA therapies, representing a characteristic limitation in the pharmacological profile of RNAi-based pharmaceuticals. In this context, prior studies have employed dose escalation analyses to achieve effective gene silencing while concurrently evaluating the incidence of side effects.

A strategy to overcome this limitation is to conduct preclinical studies that investigate the toxicity of C16 ALN-APP siRNAs and perform dose-escalation testing in various species and routes of administration, as detailed below [72]. The pharmacological profile, tolerability, and tissue distribution of C16-conjugated small interfering RNAs (C16-siRNAs) were comprehensively evaluated across multiple species and administration routes, including ICV, IT, intravitreal (IVT), and intranasal (IN) delivery. These studies demonstrated robust and durable gene silencing with an acceptable safety margin up to the highest doses tested.

Single ICV injections of C16-siRNA targeting the APP were administered to mice at 60 µg and 120 µg. These doses produced approximately 50% and 75% reductions in APP mRNA levels, respectively, persisting for several weeks. The pharmacodynamic effect was accompanied by a measurable decrease in Aβ deposition, reduced inflammatory markers, and partial normalization of behavioral and metabolic parameters in the AD CVN model [72].

In rats, IT administration of C16-siRNA was evaluated at 0.07 mg, 0.3 mg, and 0.9 mg as single doses, as well as 0.3 mg monthly for up to five doses to assess potential accumulation. The dose-dependent distribution and long tissue half-life (on the order of months) were confirmed, with sustained silencing of target transcripts throughout the spinal cord and cortical regions. In cynomolgus monkeys, single IT doses of C16-siRNA were escalated to 60 mg, with additional cohorts receiving approximately 45 mg. This regimen achieved 70–80% knockdown of target mRNA across multiple CNS regions. Maximal silencing occurred within one week, remained above 75% for approximately 2.5 months, declined to 50% by 4.5 months, and returned near baseline by 9 months. At any dose level, no test-item—related microscopic findings were observed in the brain, spinal cord, or dorsal root ganglia. Histopathological analyses using hematoxylin-eosin, Luxol Fast Blue, IBA1, and GFAP staining revealed no treatment-associated neuropathology. Doses up to 60 mg intrathecally were well tolerated under the conditions studied, although a formal no-observed-adverse-effect level (NOAEL) was not established. Furthermore, ex vivo human whole-blood assays showed no cytokine release or immune activation following exposure to C16-siRNA conjugates [72].

In NHPs, intravitreal administration of C16-siRNA targeting TTR was tested at 3 µg, 30 µg, 100 µg, and 300 µg per eye. Doses of 100–300 µg achieved > 80% reduction in TTR levels in aqueous humor for at least six months, without ocular inflammation or retinal pathology [72]. For pulmonary delivery, intranasal administration in rodents at 0.3–10 mg/kg produced up to 57% reduction in Sod1 mRNA at the 10 mg/kg dose, with gene silencing maintained for approximately two months. Systemic intravenous administration required substantially higher doses (≈30 mg/kg) to yield weaker and shorter-lived effects, underscoring the efficiency of localized delivery [72].

Across all species and routes, C16-siRNA exhibited a favorable tolerability profile, characterized by the absence of inflammatory or degenerative findings and minimal systemic exposure outside the target tissues. The compound demonstrated a sustained silencing for weeks to months following a single administration, suggesting potential for infrequent dosing in therapeutic applications. Nevertheless, tissue accumulation upon repeat dosing was observed in the rat CNS (monthly 0.3 mg IT regimen), highlighting the need for long-term studies on accumulation kinetics and clearance [72]. Variability in exposure following IT or ICV injection also warrants attention in translational development.

In summary, C16-siRNA achieved potent and durable gene silencing in the CNS, ocular, and pulmonary tissues across animal models, with no dose-limiting toxicity observed up to 60 mg IT in NHPs. These findings support the continued clinical development of C16-siRNA conjugates as a versatile platform for tissue-targeted RNAi therapeutics.

## 8. Conclusions and Perspectives

Advancements in therapeutic siRNAs, particularly ALN-APP, represent a significant step forward in strategies for treating neurodegenerative diseases, notably AD. The approval of patisiran in 2018 set an important precedent, while the development of ALN-APP showcases innovative strategies to overcome the challenges of delivering siRNAs to the CNS. The IT is particularly critical as it allows direct access to the CSF, facilitating effective delivery to target neurons that are otherwise difficult to transfect with nucleic acids. Historically, anatomical barriers have limited the effectiveness of available treatments; however, ALN-APP presents a promising alternative.

This specific siRNA formulation targets the reduction of beta-amyloid protein production, which is a key contributor to the pathogenesis of AD. As a C16-siRNA, ALN-APP incorporates a 16-carbon chain to enhance specificity and extend its therapeutic effects, building on the successes of earlier FDA-approved therapies. Preclinical studies conducted in various animal models, including rats, mice, and NHPs, have indicated its efficacy in reducing critical biomarkers related to amyloid production.

The positive outcomes from the Phase 1 trial, ALN-APP-001, further highlight this potential, revealing reductions in sAPP levels that indicate meaningful impacts on the molecular pathology of the disease. Importantly, reductions of 55% in sAPPα and 69% in APPβ by day 15, along with sustained effects across patient cohorts for up to six months, suggest that ALN-APP may represent a promising disease-modifying therapy for slowing AD progression.

Moreover, ALN-APP’s favorable safety profile enhances its suitability as a therapeutic option, suggesting its potential for long-term use in patients with EOAD. As research progresses, the insights gained from studies involving ALN-APP will be crucial for refining siRNA delivery methods and developing effective therapeutic strategies for neurodegenerative diseases.

In summary, C16 ALN-APP siRNAs represent a promising advancement in RNAi technology, particularly for treating CNS diseases, where brain tissues and neuronal cells present inherent barriers to nucleic acid transfection. These engineered siRNAs may be regarded as having crossed the “Death Valley” that separates scientific findings from clinical application. One key advantage is the absence of a delivery particle, which is addressed by the C16 tail—an essential molecular modification of ALN-APP siRNAs.

New challenges will arise in this context, whether completing the Phase 1 trials in 2029 confirms preliminary efficacy and tolerability or whether further studies demonstrate slowing disease progression in EOAD. The first challenge is to make this RNAi therapy accessible to a larger proportion of EOAD patients, particularly regarding the cost of treatment, as doses of 50 mg of this engineered siRNA will be required with re-injection every 6 or 12 months.

The second challenge involves scalability, as the large-scale production of ALN-APP siRNAs necessitates dedicated infrastructure due to the complexity of RNA synthesis and the associated chemical modifications. Lastly, specialized services are needed to administer the oligonucleotides via the intrathecal route, which, while more invasive than intravenous administration, is less invasive than the intracerebroventricular route also used for gene therapy delivery. Addressing these challenges will be important as we progress in the clinical applications of RNAi therapies.

In conclusion, the ongoing advancement of ALN-APP enhances our understanding of siRNA technology and holds significant promise for the treatment of EOAD. This development marks a significant progression in the field of RNAi therapeutics for neurological disorders.

## Figures and Tables

**Table 1:** Comparative summary of siRNA delivery strategies.

siRNA Modification	Delivery Mechanism	Target Organ/Cells	Administration Route	Advantages	Disadvantages
LNPs	Encapsulated in ionizable lipid nanoparticles, coated with ApoE for hepatocyte targeting via ApoE receptors	Liver/hepatocytes	IV	- Protects siRNA from degradation - FDA-approved	- Infusion under medical supervision - Complex formulation - Possible infusion-related reactions
GalNAc Conjugation	N-acetylgalactosamine bind to ASGPR on hepatocytes	Liver/hepatocytes	SC	- Eliminates need for nanoparticles - Self-administration possible - FDA-approved	- Limited use for extrahepatic diseases
C16 Conjugation (2′-O-Hexadecyl-siRNA)	C16 fatty acid chain enhances lipophilicity and membrane interactions	CNS/neurons, astrocytes, microglia; or ocular and pulmonary tissues)	IT or ICV	- Overcomes blood–brain barrier - Broad biodistribution in CNS - Durable silencing (months) - Expands siRNA use beyond liver (brain, lung, eye) - Preclinical success in neurodegenerative diseases	- Invasive administration (IT/ICV) - Limited penetration in some regions (e.g., striatum) - Complex optimization needed (chain length, attachment site)

IV, intravenous; SC, subcutaneous; IT, intrathecal; ICV, intracerebroventricular; LNPs, lipid nanoparticles. References supporting the data presented in this table: [64,65,69,70,71,72,73,74,75].

## Data Availability

No new data were created or analyzed in this study.

## Abbreviations

The following abbreviations are used in this manuscript:

AAIC	Alzheimer’s Association International Conference
aCSF	Artificial cerebrospinal fluid
AD	Alzheimer’s disease
AEs	Adverse events
AGO2 enzyme	Argonaute 2 enzyme
AHP	Acute hepatic porphyria
ALA	δ-aminolevulinic acid
ALS	Amyotrophic lateral sclerosis
ALT	Alanine aminotransferase
APP	Amyloid precursor protein
ApoE	Apolipoprotein E
ARIA-H	Amyloid-related imaging abnormalities-hemosiderin
ASGPR	Asialoglycoprotein receptor
Aβ	β-amyloid
AV	Autophagic vacuole
CAA	Cerebral amyloid angiopathy
CDR	Clinical dementia rating
CNS	Central nervous system
CSF	Cerebrospinal fluid
CVN	Cerebrovascular amyloid Nos2^−/−^, a genetically modified mouse used to study Alzheimer’s disease
DEAF1	Deformed epidermal autoregulatory factor-1
ds-siRNA	Double-stranded siRNA
EOAD	Early-onset Alzheimer’s disease
FDA	Food and Drug Administration
GalNAc	N-acetylgalactosamine
GFAP	Glial fibrillary acidic protein
GNA	Glycol nucleic acid
hATTR	Hereditary transthyretin amyloidosis
ICV	Intracerebroventricular
IHC	Immunohistochemistry
IN	Intranasal
IT	Intrathecal
IV	Intravenous
IVT	Intravitreal
LNP	Lipid nanoparticle
LRP1	Lipoprotein receptor-related protein
MAD	Multiple ascending dose
MCI	Mild cognitive impairment
MMSE	Mini-Mental State Examination
mRNA	Messenger RNA
NfL	Neurofilament light chain
NFT’s	Neurofibrillary tangles
NHPs	Non-human primates
NMDA	N-methyl D-aspartate
mAbs	Monoclonal antibodies
MSD	Meso-scale discovery
MTD	Maximum tolerated dose
PACT	Protein activator of the interferon-induced protein kinase
PBG	Porphobilinogen
PD	Parkinson’s disease
PET	Positron emission tomography
PK	Pharmacokinetics
PS	Phosphorothioate
RISC	RNA-induced silencing complex
RNAi	RNA interference
ROS	Reative oxygen species
RT-qPCR	Reverse transcriptase—quantitative polymerase chain reaction
SAD	Single ascending dose
sAPPα	Soluble APPα
sAPPβ	Soluble APPβ
siRNA	Small interfering RNA
Sod1	Superoxide dismutase 1
SR-BI	Scavenger receptor B type I
TRBP	Transactivation response RNA-binding protein
TTR	Transthyretin
TTR-FAP	Transthyretin familial amyloidotic polyneuropathy
VLDLR	Very low-density lipoprotein receptor
VP	Vinylphosphonate, metabolic stable analog phosphate modification. Added to avoid phosphatase degradation
Wt	Wild type
